# Utility of illness symptoms for predicting COVID-19 infections in children

**DOI:** 10.1186/s12887-022-03729-w

**Published:** 2022-11-10

**Authors:** Geena Y. Zhou, Nicole Y. Penwill, Grace Cheng, Prachi Singh, Ann Cheung, Minkyung Shin, Margaret Nguyen, Shalini Mittal, William Burrough, Mia-Ashley Spad, Sarah Bourne, Naomi S. Bardach, Emily R. Perito

**Affiliations:** 1 Children’s Hospital Los Angeles, 4650 Sunset Blvd, Los Angeles, CA 90027 USA; 2 Children’s Hospital Colorado, 13123 E 16Th Ave, Aurora, CO 80045 USA; 3 Palo Alto Medical Foundation, 370 Distel Circle, Los Altos, CA 94022 USA; 4 Department of Pediatrics, University of California San Francisco, 550 16th Street 4th Floor Box 0136, San Francisco, CA 94143 USA; 5 Tufts Medical Center, 800 Washington St, Boston, MA 02111 USA

**Keywords:** COVID-19, Symptoms, Screening, Testing, School, Testing and exclusion

## Abstract

**Background:**

The Centers for Disease Control and Prevention and the American Academy of Pediatrics recommend that symptomatic children remain home and get tested to identify potential coronavirus disease 2019 (COVID-19) cases. As the pandemic moves into a new phase, approaches to differentiate symptoms of COVID-19 versus other childhood infections can inform exclusion policies and potentially prevent future unnecessary missed school days.

**Methods:**

Retrospective analysis of standardized symptom and exposure screens in symptomatic children 0–18 years tested for SARS-CoV-2 at three outpatient sites April to November 2020. Likelihood ratios (LR), number needed to screen to identify one COVID-19 case, and estimated missed school days were calculated.

**Results:**

Of children studied (*N* = 2,167), 88.9% tested negative. Self-reported exposure to COVID-19 was the only factor that statistically significantly increased the likelihood of a positive test for all ages (Positive LR, 5–18 year olds: 5.26, 95% confidence interval (CI): 4.37–6.33; 0–4 year olds: 5.87, 95% CI: 4.67–7.38). Across ages 0–18, nasal congestion/rhinorrhea, sore throat, abdominal pain, and nausea/vomiting/diarrhea were commonly reported, and were either not associated or had decreased association with testing positive for COVID-19. The number of school days missed to identify one case of COVID-19 ranged from 19 to 48 across those common symptoms.

**Conclusions:**

We present an approach for identifying symptoms that are non-specific to COVID-19, for which exclusion would likely lead to limited impact on school safety but contribute to school-days missed. As variants and symptoms evolve, students and schools could benefit from reconsideration of exclusion and testing policies for non-specific symptoms, while maintaining testing for those who were exposed.

**Supplementary Information:**

The online version contains supplementary material available at 10.1186/s12887-022-03729-w.

## Introduction

School closures related to the coronavirus disease 2019 (COVID-19) pandemic impacted more than 57 million school-aged and 21 million in preschool or childcare in the United States in March 2020 [1]. In-person school instruction is again the expectation, but protocols for COVID-19 screening varied widely throughout the pandemic and the CDC still recommends testing as soon as possible if symptomatic and staying home [2, 3].

For schools and daycares, the Centers for Disease Control (CDC) and the American Academy of Pediatrics (AAP) recommend that children with COVID-19 symptoms stay home and be tested as soon as possible [3, 4]. However, symptoms of COVID-19 overlap extensively with other common childhood viral syndromes. The CDC and AAP’s lists of COVID-19 symptoms in children included non-specific symptoms that were not previously strict school exclusion criteria, like fatigue, headache, sore throat, and nasal congestion/rhinorrhea [4, 5]. One study previously reported that common symptoms like cough and rhinorrhea did not predict a positive severe acute respiratory syndrome coronavirus 2 (SARS-CoV-2) test in children [6]. In addition, despite widespread symptom screening during the COVID-19 pandemic, there is limited evidence on its efficacy for detecting COVID-19 cases [7]. It is possible that symptom screening led to missed school days but detected few COVID-19 cases, exacerbating learning losses for children. As the pandemic continues, with ongoing appearance of new variants and the continued stress of pandemic protocols on schools, approaches are needed to narrow the list of potential symptoms that require exclusion and testing, to optimize school attendance. Ideally such approaches could prevent overly general exclusions that exacerbate learning loss—and allow for updated symptom guidance based on new evidence as it becomes available, to account for changing symptom profiles with new variants – or future pandemics.

To assess the potential impact of exclusion and testing of symptomatic children on detection of COVID-19 and on school or child care days missed, we used cross-sectional multi-center outpatient data from symptomatic children and youth, similar to the general population of K-12 schools or daycares. For symptoms recommended by the CDC for exclusion and testing, we calculated positive likelihood ratios (+ LRs) to quantify the number of people needed to screen to identify potential infections and estimated the number of school days missed because of exclusion for symptoms. This quantifies the potential educational detriment to students [8, 9].

## Methods

### Study design and population

This was a cross-sectional, retrospective analysis of symptomatic children 0–18 years old who were tested for SARS-CoV-2 at three outpatient sites in Northern California, April 1 through November 30, 2020. This study was approved by the University of California San Francisco’s Committee on Human Research (CHR #20–32,287) and by the Sutter Health San Francisco Institutional Review Board (Local Board Reference #2020.168EXP). All methods were carried out in accordance with relevant guidelines and regulations. Analysis preceded outbreak of the Alpha, Delta and Omicron variants, but these variants’ pediatric symptom profile is similar to the original strain and earlier variants [10, 11].

Children tested were referred by an advice nurse or other outpatient pediatric provider for evaluation and COVID-19 testing. Given persistent PCR positivity in some individuals, we included the first positive test for each child and excluded all subsequent tests [12, 13]. Other excluded tests were those 1) with an incomplete symptom screen (Site 1: if only 1 of a 2-page screener was scanned into the patient’s record; Site 3: if < 5 symptoms were marked as present/absent); 2) with no documented symptom screen (*n* = 564); 3) done on asymptomatic children for procedures or school clearance (*n* = 219); 4) collected ≥ 7 days after symptom screen (*n* = 106) to only capture symptoms close to the time of test positivity or negativity, assuming that symptoms may have changed ≥ 7 days after the screen.

All tests utilized reverse transcription-polymerase chain reaction or transcription mediated amplification assay.

### Symptom screening

Symptom data were gathered from site-specific, standardized symptom screeners completed by a healthcare provider during an in-person or telehealth visit prior to the COVID-19 test. As slight differences in screeners existed between sites, we grouped symptoms into two categories: those on the CDC list of COVID-19 symptoms in children [4] and other symptoms potentially used for school exclusion.

#### Site 1 (Benioff Children’s Hospital Oakland)

Symptoms screened included: Fever or chills, cough, nausea/vomiting/diarrhea, dyspnea, nasal congestion/rhinorrhea, muscle aches, loss of taste or smell, conjunctivitis, and rash. COVID-19 contact exposure and rash were added to this screener on July 23, 2020; we screened charts for contacts reported prior to this date. Data was extracted by manual chart review.

#### Site 2 (Benioff Children’s Hospital San Francisco)

Symptom screen data was extracted electronically or manually from the screener in the medical record. Compared to Site 1, additional symptoms screened include abdominal pain, headache, sore throat, and fatigue.

#### Site 3 (Palo Alto Medical Foundation, San Carlos)

For feasibility of data extraction from this larger cohort, all COVID-19 positive tests were identified and then matched, by age in years and month of testing, to four COVID-19 negative controls who had no previous COVID-19 positive tests; 12 cases had only three controls available. Three were excluded for < 3 matched controls. Symptoms screened included: Fever or chills, cough, nausea/vomiting/diarrhea, dyspnea, nasal congestion/rhinorrhea, muscle aches, loss of taste or smell (added April 12, 2020), and rash. Fatigue and conjunctivitis were not screened.

### Site and county COVID-19 prevalence

To estimate prevalence in counties of residence for our study population, we used population-level county 7-day positivity for the 1st and 3rd weeks of each month from publicly available county Department of Public Health data [14–18]. For Site 3, county data was not available from the public health department or other sources; we thus used microbiology lab data from that center on 14-day positivity for children ages 0–18 tested each month (Supplemental Fig. 1).

**Fig. 1:**
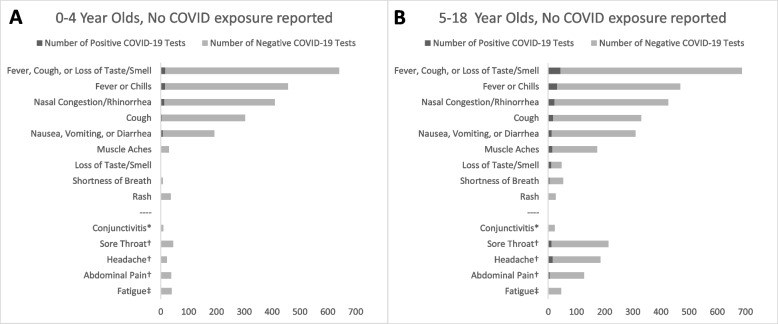
Frequency of symptoms and COVID-19 positive versus negative test result in children with no reported COVID-19 exposure, by age. *Children at Sites 1 and 2; Site 3 symptom screener did not include. †Children tested at Sites 1 and 3; Site 2 symptom screener did not include. ‡Children tested at Site 1; Sites 2 and 3 symptom screeners did not include

### Demographics

Demographics were extracted from electronic medical records. Age was categorized as 0–4, 5–18 years to reflect childcare and K-12 school-aged groups.

### Exposure to COVID-19

Recent close contact with someone who had COVID-19 was self-reported by the patient or caregiver. Data from contact tracing was not available.

### Symptoms

We combined the following symptoms for analysis: Fever, chills as “fever/chills”; sinus congestion, runny nose, rhinorrhea, nasal congestion, and chest congestion as “nasal congestion/rhinorrhea”; eye redness, eye discharge, and eye pain as “conjunctivitis”; and “nausea, vomiting, or diarrhea.”

### Health status

Health status was determined using the Pediatric Medical Complexity Algorithm Version 3.1 (SAS Institute Inc., Cary, NC) [19]. *International Statistical Classification of Diseases and Related Health Problems, Tenth Revision (ICD-10)* codes were extracted from the medical record (last three years, inpatient and outpatient encounters) or the problem list if no recent encounters (*n* = 6). Children with no chronic conditions or non-chronic complex disease were categorized as “without complex chronic disease.”

### Statistical analysis

For each symptom or symptom combination, we assessed (1) percent of children reporting the symptom, (2) positive LRs, which relate the change in probability of a positive test if an individual has the symptom [20], (3) number of individuals needed to screen for the symptom to identify one case [21]; and (4) number of missed school days to identify one case, due to isolation while waiting for a test, estimating 3 days missed for each positive symptom screen (day of symptom identification, day of testing, day waiting for results) (See Supplemental Table 1 for formulae).

**Table 1:** Children with illness symptoms tested for COVID-19, by test result, April – November 2020

Characteristic	Total *N* = 2,167	Positive COVID-19 Test *N* = 241	Negative COVID-19 Test *N* = 1926
**Age at testing in years, n (%)**			
0–4	960 (44.3%)	76 (31.5%)	884 (45.9%)
5–11	655 (30.2%)	71 (29.5%)	584 (30.3%)
12–18	552 (25.5%)	94 (39.0%)	458 (23.8%)
Median age (IQR)	^a^	9 (3–13)^b^	4 (2–9)^b^
**Sex, %**			
Female	49.1%	49.4%	49.1%
**Ethnicity, %**			
Hispanic or Latino	22.7%	49.0%	19.4%
Unknown/Declined	19.7%	15.8%	20.1%
**Race, %**			
White	36.6%	26.1%	38.0%
Asian	11.9%	8.3%	12.4%
Black or African American	5.0%	4.1%	5.1%
Other	25.0%	42.3%	22.8%
Unknown/Declined	21.4%	19.1%	21.7%
**Language Preference, %**			
English	92.4%	82.2%	93.7%
Spanish	6.0%	16.6%	4.7%
Other	1.6%	1.2%	1.6%
**Without complex chronic disease, %**	83.7%	83.8%	83.7%
**Known Contact Exposure, %**	19.1%	70.1%	12.7%

^a^Unable to calculate, based on Site 3 1:4 case–control matching by age and COVID test result date

^b^Sites 1 and 2 only; Site 3 not included because of case–control matching by age

We examined each symptom individually. In addition, we examined combinations of symptoms (1) that have been more specifically associated with COVID-19 infection (e.g. fever, cough, loss of taste/smell) or (2) that are less specific to COVID-19 but are common childhood symptoms and are included in the CDC list of COVID-19 symptoms [2, 3].

To assess the clinical relevance of the calculated LRs, we used an LR nomogram; this illustrates the probability of testing COVID-19 positive in a child with that symptom (post-test probability) for select symptoms and symptom combinations [22]. We used a pre-test probability of 5%, based on community prevalence during the study period and the CDC indicator for low to moderate transmission risk for school decision-making [3].

We conducted 2 sensitivity analyses. Because the sampling scheme was different between sites, we calculated LRs for Sites 1 and 2 separately from Site 3. We also used logistic regression to calculate adjusted odds ratios for each symptom [23], adjusting for site and COVID-19 exposure. For the adjusted logistic regression models, we were unable to combine person-level data from Site 3 with that from Sites 1 and 2 due to data-sharing limitations between institutions.

We used Stata 16 (StataCorp, College Station, TX) for statistical analyses [24].

## Results

The study population included 2,167 children; 83.7% were without complex chronic medical conditions, and 88.9% tested negative. Almost 20% reported a recent exposure to COVID-19 (Table 1). Across counties of residence for included children, percent positivity of COVID-19 tests ranged from 0.8 to 9.8% during the study period and was higher in some counties than others (Supplemental Fig. 1).

The most frequently reported symptoms were “fever/chills” (*n* = 1080, 49.8%) and “nasal congestion/rhinorrhea” (*n* = 1023, 47.2%). Over two-thirds of all children reported > 1 illness symptom, including 69.3% of COVID-19 positive and 68.4% of COVID-19 negative children. Amongst children with no reported COVID-19 exposure, the vast majority – including those with fever/chills, cough, or loss of taste or smell – tested negative (Fig. 1). For those children with reported exposure, the percent positive was higher across all symptoms compared to those without exposure (Fig. 1, Supplemental Fig. 2).

**Fig. 2:**
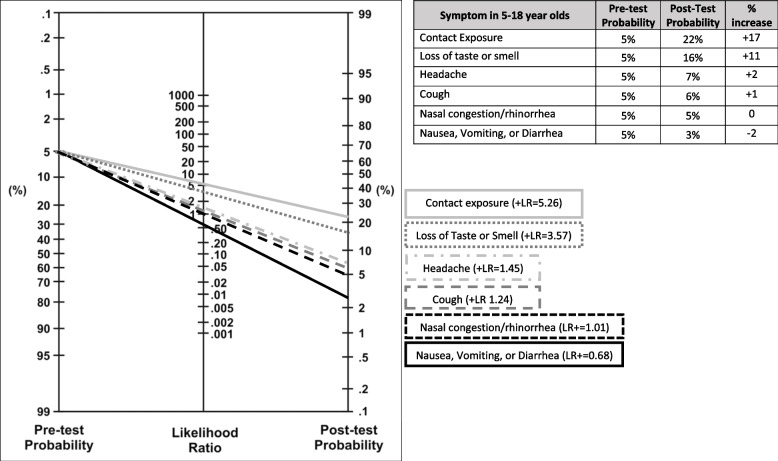
Impact of common illness symptoms on the probability of testing COVID-19 positive. The Fagan nomogram is a graphical tool that demonstrates the probability that a child with each symptom has a positive COVID-19 test, in a community with 5% prevalence (pre-test probability). Identifying contact exposure or loss of taste or smell in a symptom screener identifies children with a higher probability of actually having a COVID-19 infection (post-test probability) than a randomly selected child from the community. In contrast, identifying symptoms with LR 1–2 do not meaningfully increase the probability that a tested child will have COVID-19 above the background community prevalence

### Factors associated with a COVID-19 positive test

Reported exposure to someone with COVID-19 was the only factor with a statistically significantly positive likelihood ratio for all ages (Table 2).

**Table 2:** Utility of symptoms identified on symptom screener for identifying COVID cases in children with illness symptoms (*n* = 2167)

		**% of cohort with this symptom**	**Positive Likelihood Ratio (95% CI)**	**Number Needed to Screen**	**Estimated missed school days**
			**Among children with illness symptoms, how much does having this symptom change the likelihood of testing positive for COVID-19?**	**How many children with illness symptoms need to be screened for this symptom to find 1 case of COVID-19?**	**How many school days will be missed by children with this symptom** **to find 1 case of COVID-19?**
**In 5–18 year olds (*n* = 1207), any presence of**					
CDC-listed COVID-19 symptoms for school exclusion and immediate testing [2, 3, 5]	Fever or chills	46.1%	1.11 (0.94–1.31)	15	20
	Sore Throat^a^	42.7%	1.00 (0.78–1.29)	15	19
	Headache^a^	39.3%	1.45 (1.16–1.80)*	12	14
	Cough	36.3%	1.24 (1.03–1.51)*	17	18
	Nausea, Vomiting, or Diarrhea	30.0%	0.68 (0.50–0.92)*	34	31
	Shortness of Breath	5.0%	1.26 (0.65–2.44)	121	18
	Nasal Congestion/Rhinorrhea	43.7%	1.01 (0.84–1.22)	17	22
	Fatigue^b^	34.5%	2.17 (1.29–3.63)*	35	36
	Muscle Aches	18.7%	1.44 (1.08–1.93)*	29	16
	Loss of taste or smell	6.0%	3.57 (2.27–5.61)*	46	8
Other illness symptoms potentially used for school exclusion	Conjunctivitis^c^	3.7%	1.09 (0.34–3.53)	256	21
	Rash||	3.1%	0.20 (0.03–1.43)	1055	99
	Abdominal Pain^a^	20.7%	0.56 (0.32–0.98)*	51	32
Self-reported COVID-19 contact^d^		24.0%	5.26 (4.37–6.33)*	9	7
**In 0–4 year olds (*n* = 960), any presence of**					
CDC-listed COVID-19 symptoms for school exclusion and immediate testing	Fever or chills	54.6%	1.31 (1.11–1.54)*	18	30
	Cough	38.6%	0.77 (0.54–1.09)	42	48
	Nausea, Vomiting, or Diarrhea	23.5%	0.77 (0.47–1.25)	69	48
	Sore Throat^a^	12.7%	1.40 (0.70–2.77)	55	21
	Headache^a^	5.9%	0.70 (0.17–2.86)	221	39
	Shortness of Breath	1.0%	2.91 (0.63–13)	480	15
	Nasal Congestion/Rhinorrhea	51.6%	1.05 (0.85–1.31)	23	36
	Fatigue^b^	20.2%	1.37 (0.51–3.73)	86	52
	Muscle Aches	3.3%	1.66 (0.60–4.61)	240	24
	Loss of taste or smell	0.1%	0.00 (0.16–93)	#	#
Other illness symptoms potentially used for school exclusion and testing	Rash^d^	4.7%	0.92 (0.29–2.91)	291	41
	Conjunctivitis^c^	1.7%	1.54 (0.20–12)	777	39
	Abdominal Pain^a^	9.8%	0.63 (0.20–1.95)	147	43
Self-reported COVID-19 contact^d^		18.4%	5.87 (4.67–7.38)*	16	9

Includes children from all 3 sites combined, with and without reported COVID exposure. Assumes that each child sent for COVID-19 testing misses 3 days of school, for PCR testing.

*CI *  Confidence Interval

^a^Includes only children tested at Site 2 and 3; Site 1 symptom screener did not include

^b^Includes only children tested at Site 2; Sites 1 and 3 symptom screeners did not include

^c^Includes only children tested at Site 1 and 2; Site 3 symptom screener did not include

^d^Symptom added to Site 1 screener during study, number needed to screen excludes children not screened for symptom

^*^p < 0.05

In 5–18 year olds, of the CDC symptoms recommended for school exclusion [2, 3, 5], headache, cough, fatigue, muscle aches, and loss of taste or smell had statistically significant positive likelihood ratios (Table 2). However, the positive likelihood ratios for headache, cough, and muscle aches were less than 2, thus resulting in minimal changes to post-test probability (Fig. 2); a substantial number of school days would be missed to identify one case (14, 18, 16 respectively). Loss of taste or smell, though rare (6.0%), had a high and statistically significant positive likelihood ratio (LR: 3.57 95% CI 2.27–5.61) and substantially changed post-test probability (+ 11 percentage points) (Fig. 2).

The most common symptoms, fever/chills (46.1%), nasal congestion (43.7%), and sore throat (42.7%), had positive likelihood ratios close to 1.00, none of which were statistically significant (Table 2).

All other symptoms in both age groups (0–4 and 5–18) were either not meaningfully associated with a positive likelihood of COVID-19 (LR of < 2), were not statistically significantly associated, or had a significantly decreased association (e.g., nausea, vomiting or diarrhea; LR: 0.68, 95% CI 0.50–0.92, in 30% of 5–18 year olds). This included nasal congestion, which was very common in both groups (43.7% of 5–18 and 51.6% in 0–4) (Table 2).

In our assessment of co-occurring symptoms, in 5–18 year old children without fever, only cough and loss of taste or smell remained significantly associated with COVID-19 positivity (Table 3). In the absence of fever or cough, none of the following symptoms increased the likelihood of testing COVID-19 positive: sore throat, nausea/vomiting/diarrhea, fatigue, abdominal pain, headaches, muscle aches, shortness of breath (Table 3). Isolating and testing every 5–18 year old with no fever and only one of the following symptoms would lead to > 18 days of school missed per COVID-19 infection identified: nasal congestion/rhinorrhea, sore throat, nausea/vomiting/diarrhea, fatigue, abdominal pain, and muscle aches (Table 3).

**Table 3:** Diagnostic utility of common symptom combinations in children 5–18 years old with illness symptoms who were tested for COVID-19^a^

**Symptom combinations** *–* = *Symptom absent* + = *Symptom present* *Blank* = *Symptom not considered in this combination*					**% with this symptom combination**	**Positive** **Likelihood Ratio** **(95% CI)**	**Number Needed to Screen to find 1 COVID-19 case**	**Estimated Missed School Days to find** **1 COVID-19 case**
**Fever or chills**	**Cough**	**Congestion/Rhinorrhea**	**Loss of Taste/Smell**	**Other Symptoms**				
Fever or chills	Cough	Congestion/Rhinorrhea	Loss of taste/smell		0.5%	6.32 (1.29–31)†	352	6
Fever or chills	–	–	–		23.2%	0.67 (0.47–0.97)†	39	31
–	Cough	–	–		8.0%	1.74 (1.11–2.75)†	50	14
–	–	–	Loss of taste/smell		1.1%	3.95 (1.31–12)†	211	8
–	–	Congestion/Rhinorrhea	–		14.0%	1.20 (0.82–1.75)	39	19
Fever or chills	–	Congestion/Rhinorrhea	–		7.5%	0.97 (0.54–1.74)	88	23
–	Cough	Congestion/Rhinorrhea	–		13.5%	0.41 (0.22–0.77)†	106	49
–	–		–	Sore Throat^b^	14.0%	0.48 (0.23–1.00)	89	38
–	–		–	Nausea, Vomiting, or Diarrhea	10.2%	0.32 (0.14–0.72)†	175	61
–	–		–	Fatigue^c^	8.0%	1.84 (0.28–12)	175	42
–	–		–	Abdominal Pain^b^	6.7%	0.43 (0.13–1.35)	214	43
–	–		–	Headache^b^	5.9%	0.55 (0.22–1.37)	102	12
–	–		–	Muscle Aches	4.9%	0.85 (0.39–1.84)	150	25
–	–		–	Shortness of breath	0.5%	0.00 (0.04–12)	||	||

Includes children from all 3 sites combined, with and without reported COVID exposure. “Fever or chills” listed as “fever.” Assumes that each child sent for COVID-19 testing misses 3 days of school, for PCR testing. If only 1 day school missed per child, then estimated missed school days = number needed to screen

|| Number Needed to Screen unreportable; no one with this symptom tested positive

^a^Children may have had additional symptoms reported that are not considered in this summary

^b^Includes only children tested at Site 2 and 3; Site 1 symptom screener did not include

^c^Includes only children tested at Site 2; Sites 1 and 3 symptom screeners did not include

^†^*p* < 0.05

### Associations with COVID-19 positivity, adjusted for site and reported exposure

In sensitivity analyses of results by site, the only factors consistently associated with COVID-19 positivity across sites were COVID-19 exposure (all ages) and loss of taste/smell for 5–18 year olds (Supplemental Tables 2, 3). Fever/chills for 0–4 year olds increased the odds of being COVID-19 positive in analyses adjusted for contact exposure (Supplemental Table 3).

## Discussion

This multi-site study of symptomatic children evaluated in outpatient settings during the COVID-19 pandemic demonstrates an approach that could be repeated systematically to inform school exclusion and testing policies and practices. Our study population was likely similar to those with symptoms at school or childcare: most with no reported COVID-19 exposure; few with complex medical conditions; and all tested as outpatients, so likely mildly ill. The majority of these symptomatic children tested negative for COVID-19. We found that, of the eleven COVID-19 symptoms that the CDC suggests should trigger COVID-19 testing [2, 3, 5], nasal congestion, sore throat, and “nausea, vomiting, and diarrhea” were very common (in 30–50% of children) and not significantly associated with a positive COVID-19 test in any age group; they were, however, associated with substantial missed school-days. Loss of taste or smell was strongly associated with increased likelihood of a positive test, as was self-reported COVID-19 exposure. These data suggest that, in an endemic phase of the COVID-19 pandemic with ongoing new variants [25, 26], more parsimonious lists of symptoms for exclusion might be appropriate.

Our findings that (1) COVID-19 exposure was the most significant predictor of having COVID-19, (2) other non-specific symptoms of illness either were not associated with or even reduced the likelihood of testing positive for COVID-19, and (3) loss of taste or smell was a useful predictor, are similar to findings from other studies of pediatric cohorts during the same time period, including in Italy and Canada [6, 27, 28]. As circulating COVID-19 variants change, symptom frequency may also change – and re-examining pediatric symptoms over time will be required. The use of likelihood ratios, as in this study, to help demonstrate the utility of focusing on certain symptoms could be re-utilized as the disease itself evolves.

Our analysis also aimed to quantify the impact of symptoms on school days missed, a key policy consideration in light of extensive learning losses sustained, exacerbations of educational inequities due to missed school, and the detrimental effects of hybrid and distance learning [29, 30]. We found, for example, that exclusion and non-rapid, molecular testing of those with sore throat could lead to more than 35 days of school missed for one case of COVID-19 detected, as could exclusion and testing of children with most symptoms, in the absence of fever and cough. To place that number in context, California defines truancy as > 30 min of unexcused school three times per year [31]. While illness is an excused absence, this definition highlights the importance of 35 missed school days, even spread across a group of students. Again as COVID-19 variants evolve, these calculations may need to be re-visited; but our analysis offers a simple technique for quantifying one aspect of the risks associated with school exclusions or closures: missed school days.

It is important to note that our data included infections associated with the original strain and early variants, preceding the Alpha variant B.1.1.7, Delta B.1.617.2, and Omicron B.1.1.529 variants. A prospective UK study comparing symptoms in children with Alpha and Delta variant infections found that the seven most common symptoms were the same between the two variants [10]. However, studies of the Omicron variant revealed a lower incidence of loss of smell and a higher incidence of sore throat [32, 33]. Future research with new variants could improve precision of symptom-specific point estimates, though findings from older strains could inform policymakers’ approach. For instance, these data suggests that policymakers could have recommended against school exclusion for isolated rhinorrhea, since those symptoms overlap with symptoms of common coronaviruses and rhinoviruses and have not been shown predictive of COVID-19 disease [34, 35]. Westbrook et al. found a positive predictive value of only 9% for isolated congestion/rhinorrhea, even despite their cohort’s high community COVID-19 positivity (21%) during the height of the delta variant [36]. Though recent variants (e.g., Omicron) have demonstrated increasing transmissibility [37, 38], it is critical to remember that higher community prevalence (pre-test probability) does not meaningfully change the probability that children with a symptom whose LR is not significantly different than 1 will test positive for COVID-19 (post-test probability) (Fig. 2). Policymakers uncomfortable changing symptom recommendations could consider recommending rapid antigen tests for symptomatic children in a test-to-stay strategy. Antigen tests are less sensitive than molecular tests (e.g., PCR) but would minimize days missed in the setting of low pre-test probability for non-specific symptoms [39].

Our data may also inform decisions of what criteria to maintain for potential exclusion and testing. Our data supports inquiries regarding COVID-19 contact exposure in all ages and loss of taste or smell for 5–18 year olds.

This study has limitations. Although a standardized symptom screener was used at each site, the checklist, administering healthcare provider, and ambulatory setting differed slightly between sites. However, parental or school nurse assessments of symptoms will also likely vary; our findings may thus reasonably reflect real-world settings. The accuracy of symptom screening in children 0–4 was limited to caregiver and health provider observation; however, this again reflects real-world screening. In addition, we assumed that symptoms not marked on the screener as “present” were “absent.” Since we were more confident about absence of “major” symptoms like fever and cough than less specific symptoms, we limited our analysis to reflect this. For Site 3, we also excluded screeners with fewer than 5 symptoms marked present or absent to ensure more accuracy of our assumptions. Further, COVID-19 exposure was not defined across sites; it was not possible to discriminate exposure by close versus causal contacts. In subsequent studies, it will be helpful to gather information regarding whether exposure was at school or home. Finally, we may have been underpowered to detect statistically significant likelihood ratios for some symptoms that were less common. However, point estimates were close to one for most LRs that were not statistically significant, suggesting that even with a narrow confidence interval the change to post-test probability would be minimal. In addition, COVID-19 positivity was higher at our testing sites than in surrounding communities, likely due to a relatively high threshold for testing in earlier months. As testing availability has increased and threshold for testing decreased, we would expect that children with even milder or less specific symptoms might be added to the testing pool; this would likely further decrease symptom utility for identifying COVID-19 cases. To further assess the feasibility and utility of limiting school exclusion based on mild non-specific symptoms, future studies could repeat the analysis using data from more recent variants of COVID-19. Additional data on timing of symptom onset and days of school missed due to testing could inform symptomatic testing recommendations.

## Conclusion

In a large population of symptomatic children April-November 2020, the presence of most symptoms did not meaningfully increase the likelihood of testing COVID-19 positive, especially in children with no known COVID-19 exposure. This suggests that a more limited symptom list could be used as the pandemic continues to evolve – or future pandemics emerge – to create more parsimonious symptom criteria for exclusion and testing. Excluding students or staff with non-specific symptoms was unlikely to effectively or efficiently identify children with COVID-19 and likely contributed to unnecessary learning loss. The use of LRs and numbers needed to screen demonstrated that excluding and testing 5–18 year olds with loss of taste or smell, and those of all ages with exposure to COVID-19, would have been reasonable approaches to identify COVID-19 cases during the study period.

## Supplementary Information

**Additional file 1.** Supplemental figures.

**Additional file 2. **Supplemental tables.

## Data Availability

The datasets used and/or analyzed during the current study are available from the corresponding author on reasonable request.

## Abbreviations

COVID-19: Coronavirus disease 2019
CDC: Centers for Disease Control and Prevention
AAP: American Academy of Pediatrics
SARS-CoV-2: Severe acute respiratory syndrome coronavirus 2
LR: Likelihood ratio ()
