# Supplementary material for: Utility of illness symptoms for predicting COVID-19 infections in children
Source: BMC Pediatr. 2022 Nov 10;22:655. doi: 10.1186/s12887-022-03729-w (PMC9647749; doi:10.1186/s12887-022-03729-w)
Supplement: Supplementary file 1 — Additional file 1. Supplemental figures. [file 12887_2022_3729_MOESM1_ESM.pdf]

Supplemental Figure 1. Prevalence of COVID-19 positive tests among all tested, by month.

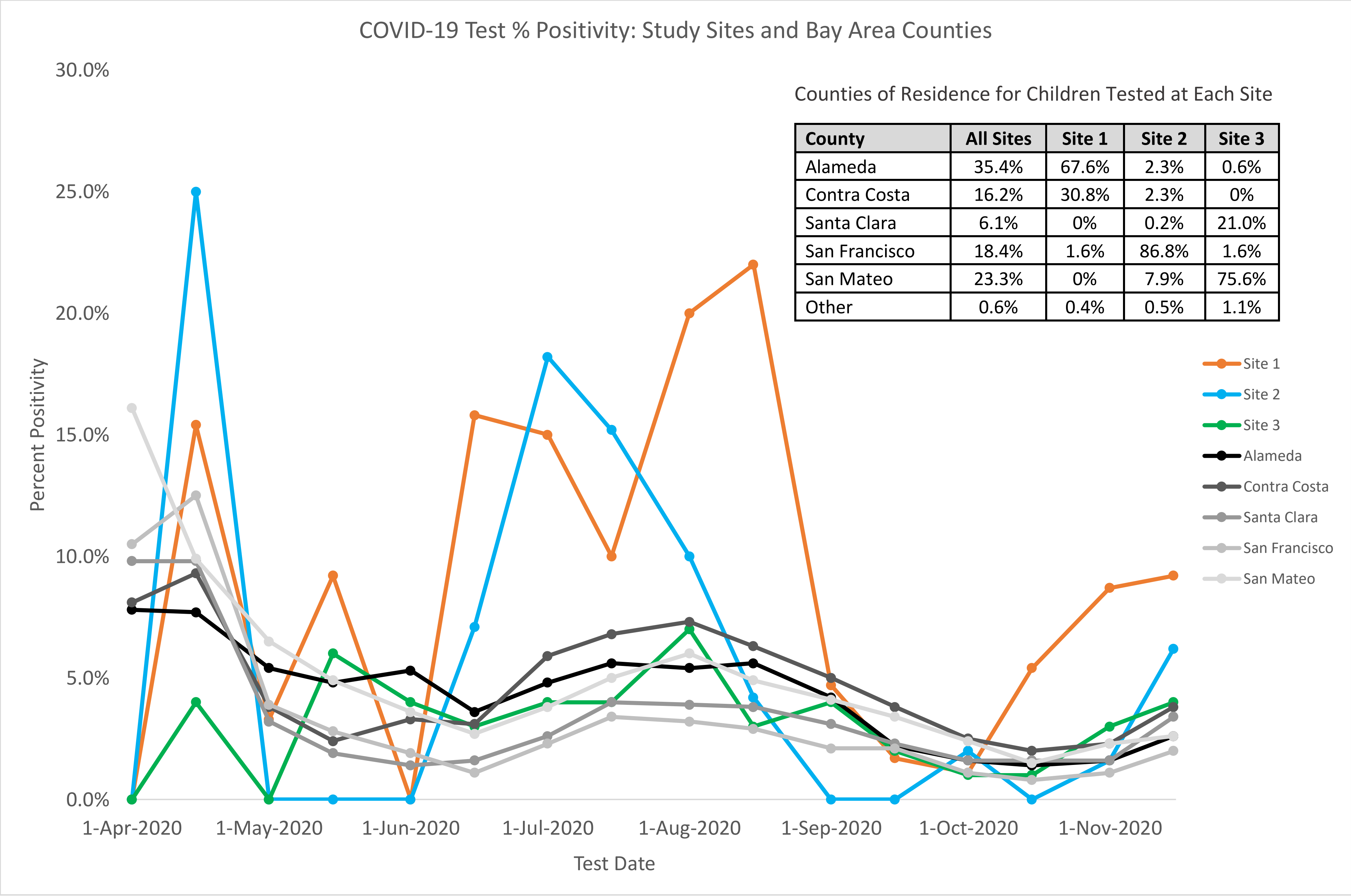

County	All Sites	Site 1	Site 2	Site 3
Alameda	35.4%	67.6%	2.3%	0.6%
Contra Costa	16.2%	30.8%	2.3%	0%
Santa Clara	6.1%	0%	0.2%	21.0%
San Francisco	18.4%	1.6%	86.8%	1.6%
San Mateo	23.3%	0%	7.9%	75.6%
Other	0.6%	0.4%	0.5%	1.1%

Supplemental Figure 2. Frequency of symptoms and COVID-19 positive versus negative test result in children with reported COVID-19 exposure by age.

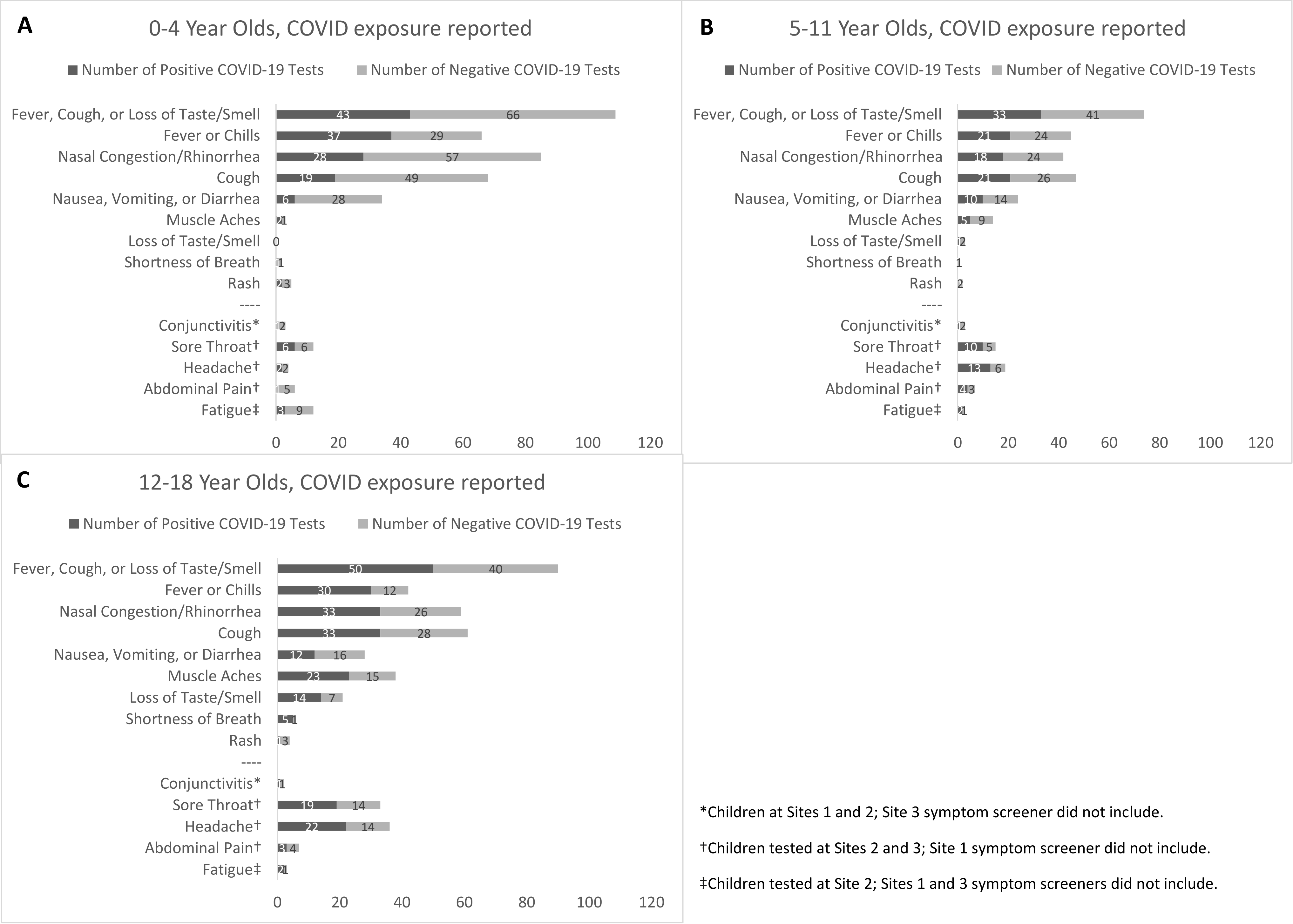

*Children at Sites 1 and 2; Site 3 symptom screener did not include.

†Children tested at Sites 2 and 3; Site 1 symptom screener did not include.

‡Children tested at Site 2; Sites 1 and 3 symptom screeners did not include.

Supplemental Figure 3. Frequency of symptoms and COVID-19 positive versus negative test result in children with no reported COVID-19 exposure, by age.

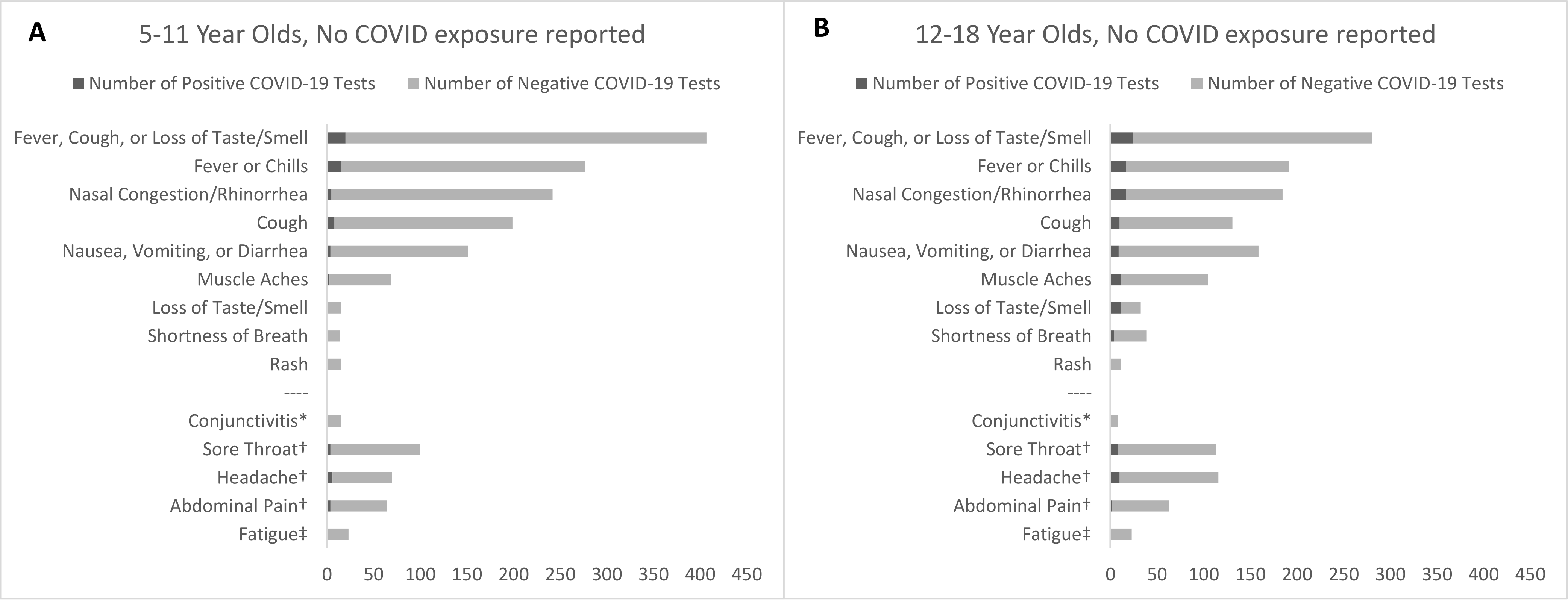

*Children at Sites 1 and 2; Site 3 symptom screener did not include.

†Children tested at Sites 2 and 3; Site 1 symptom screener did not include.

‡Children tested at Site 2; Sites 1 and 3 symptom screeners did not include.
