# Supplementary material for: Utility of illness symptoms for predicting COVID-19 infections in children
Source: BMC Pediatr. 2022 Nov 10;22:655. doi: 10.1186/s12887-022-03729-w (PMC9647749; doi:10.1186/s12887-022-03729-w)
Supplement: Supplementary file 2 — Additional file 2. Supplemental tables. [file 12887_2022_3729_MOESM2_ESM.docx]

| **Supplemental Table 1: Methods for calculating reported metric** | | |
| --- | --- | --- |
| **Metric** | **Calculation** | **Interpretation of metric** |
| Positive likelihood ratio | = True positive / False positive = sensitivity/(1-specificity) | How much does having this symptom change the likelihood of testing positive for COVID-19? |
| Negative likelihood ratio | = False negative / True negative = (1 – sensitivity) / specificity | How much does NOT having this symptom change the likelihood of testing positive for COVID-19? |
| Pre-test odds | = Pretest probability / (1 − Pretest probability) | What are the odds the child has COVID-19 prior to testing? |
| Post-test odds | = Pretest odds × Likelihood ratio | What are the odds the child has COVID-19 after testing results are known? |
| Post-test probability | = Posttest odds / (1 + Posttest odds) | How likely does a child have COVID-19 after testing is performed? |
| Number needed to screen | # of children screened for symptom /# with symptom or symptom combination and positive for COVID-19 | How many children with that symptom need to be screened to identify 1 case of COVID-19? |
| Missed school days | (# reporting the symptom x 3 school days missed) /# COVID-19 cases identified in children with that symptom | How many missed school days would be caused by screening for that symptom, and requiring isolation and testing for every child with that symptom? |

| **Supplemental Table 2: Impact of illness symptoms on COVID-19 positivity in symptomatic children tested for COVID-19, April – November 2020** | | | |
| --- | --- | --- | --- |
| **Metric** | | **Site 1, 2**  **Positive Likelihood Ratio (95% CI)** | **Site 3**  **Positive Likelihood Ratio (95% CI)** |
|  | | **Among children with illness symptoms, how much does having this symptom change the likelihood of testing positive for COVID-19 infection?** | |
| **In 5-18 year olds, any presence of** | | | |
| CDC-listed pediatric COVID-19 symptoms ^2,4^ | Fever or chills | 1.06 (0.84-1.34) | 1.21 (0.95-1.53) |
|  | Sore Throat† | 2.06 (1.23-3.43)* | 0.87 (0.66-1.15) |
|  | Headache† | 1.40 (0.58-3.40) | 1.36 (1.08-1.72)* |
|  | Cough | 1.15 (0.87-1.51) | 1.48 (1.11-1.97)* |
|  | Nausea, Vomiting, or Diarrhea | 0.55 (0.32-0.94)* | 0.70 (0.48-1.03) |
|  | Shortness of Breath | 0.79 (0.19-3.29) | 1.17 (0.55-2.48) |
|  | Nasal Congestion/Rhinorrhea | 0.90 (0.68-1.19) | 1.17 (0.91-1.51) |
|  | Fatigue‡ | 2.17 (1.29-3.63)* | \|\| |
|  | Abdominal Pain† | 0.77 (0.12-4.86) | 0.52 (0.29-0.93)* |
|  | Muscle Aches | 1.12 (0.68-1.84) | 1.58 (1.09-2.29)* |
|  | Loss of taste or smell | 3.37 (1.70-6.69)* | 3.32 (1.78-6.19)* |
| Other illness symptoms potentially used for school exclusion | Conjunctivitis§ | 1.09 (0.34-3.53) | \|\| |
|  | Rash | 0.00 (0.01-3.42) | 0.36 (0.05-2.74) |
| Self-reported COVID-19 contact exposure | | 4.33 (3.41-5.50)* | 7.77 (5.48-11)* |
| **In 0-4 year olds, any presence of** | | | |
| CDC-listed pediatric COVID-19 symptoms ^2,4^ | Fever or chills | 1.19 (0.91-1.55) | 1.20 (0.98-1.46) |
|  | Cough | 0.43 (0.22-0.84)* | 1.39 (0.90-2.15) |
|  | Nausea, Vomiting, or Diarrhea | 0.65 (0.31-1.38) | 0.76 (0.39-1.47) |
|  | Sore Throat† | 1.55 (0.42-5.68) | 1.29 (0.56-2.99) |
|  | Headache† | 3.21 (0.43-24) | 0.24 (0.03-1.75) |
|  | Shortness of Breath | 0.00 (0.09-29) | 2.72 (0.47-16) |
|  | Nasal Congestion/Rhinorrhea | 0.98 (0.72-1.33) | 1.38 (0.97-1.97) |
|  | Fatigue‡ | 1.37 (0.51-3.73) | \|\| |
|  | Abdominal Pain† | 0.00 (0.04-9.24) | 0.53 (0.17-1.68) |
|  | Muscle Aches | 1.60 (0.39-6.56) | 1.63 (0.33-8.08) |
|  | Loss of taste or smell | 0.00 (0.25-0.45) | # |
| Other illness symptoms potentially used for school exclusion | Rash | 0.00 (0.02-4.74) | 1.53 (0.43-5.48) |
|  | Conjunctivitis§ | 1.54 (0.20-12) | \|\| |
| Self-reported COVID-19 contact exposure | | 5.47 (4.10-7.30)* | 6.89 (4.18-11)* |
| *p<0.05  † Includes only children tested at Site 2 and 3; Site 1 symptom screener did not include.  ‡ Includes only children tested at Site 2; Sites 1 and 3 symptom screeners did not include.  § Includes only children tested at Site 1 and 2; Site 3 symptom screener did not include.  \|\| LR unreportable; symptom not on screener.  # LR unreportable; all children with this symptom tested COVID-19 negative. | | | |

| **Supplemental Table 3: Association of symptoms with COVID-19 positivity, adjusted for site and reported exposure to COVID-19 contact** | | |
| --- | --- | --- |
| **Symptom** | **Site 1, 2**  **Odds Ratio (95% CI) , adjusted for site and contact exposure** | **Site 3**  **Odds Ratio (95% CI), adjusted for contact exposure** |
| **In 5-18 year olds, any presence of** |  |  |
| Fever or chills | 1.45 (0.86-2.45) | 4.72 (2.16-10.34)* |
| Sore Throat† | 6.25 (0.77-50.26)* | 1.01 (0.55-1.84) |
| Headache† | 1.50 (0.25-8.98) | 2.12 (1.15-3.89)* |
| Cough | 1.06 (0.62-1.81) | 2.30 (1.21-4.37)* |
| Nausea, vomiting, or diarrhea | 0.57 (0.29-1.13) | 0.82 (0.43-1.54) |
| Shortness of Breath | 1.32 (0.31-5.56) | 1.90 (0.62-5.8) |
| Nasal Congestion/Rhinorrhea | 1.03 (0.60-1.74) | 1.76 (0.96-3.26) |
| Fatigue§ | 6.25 (0.78-50.26)* | # |
| Abdominal Pain† | 1.00 (0.08-12.05) | 1.16 (0.49-2.74) |
| Muscle Aches | 1.16 (0.60-2.24) | 1.83 (0.94-3.57) |
| Loss of taste or smell | 3.81 (1.70-8.56)* | 2.53 (1.11-5.75)* |
| Conjunctivitis‡ | 1.41 (0.35-5.60) | # |
| Rash | \|\| | 0.44 (0.01-17.4) |
| **In 0-4 year olds, any presence of** |  |  |
| Fever or chills | 2.50 (1.24-5.03)* | 4.88 (1.71-13.96)* |
| Cough | 0.25 (0.10-0.60)* | 1.53 (0.58-4.01) |
| Nausea, vomiting, or diarrhea | 0.63 (0.26-1.53) | 0.71 (0.29-1.72) |
| Sore Throat† | 3.36 (0.42-27.11) | 0.80 (0.28-2.26) |
| Headache† | 11.99 (0.15-953.53) | 0.15 (0.01-2.55) |
| Shortness of Breath | \|\| | 1.77 (0.35-9.05) |
| Nasal Congestion/Rhinorrhea | 1.00 (0.51-1.94) | 1.97 (0.77-5.05) |
| Fatigue§ | 0.88 (0.18-4.28) | # |
| Abdominal Pain† | \|\| | 0.84 (0.23-3.02) |
| Rash | \|\| | 1.61 (0.30-8.55) |
| Conjunctivitis‡ | 1.25 (0.11-14.37) | # |
| Muscle Aches | 2.70 (0.50-14.58) | 4.45 (0.37-53.13) |
| Loss of taste or smell | \|\| | - |
| *p<0.05  † Includes only children tested at Site 2 and 3; Site 1 symptom screener did not include.  ‡ Includes only children tested at Site 2; Sites 1 and 3 symptom screeners did not include.  § Includes only children tested at Site 1 and 2; Site 3 symptom screener did not include.  \|\| Cannot calculate LR for this group, as all children with this symptom tested COVID-19 negative.  # LR unreportable; symptom not on screener. | | |

| **Supplemental Table 4: Impact of NOT having each symptom on COVID-19 test positivity in children tested for COVID-19, April – November 2020** | | | |
| --- | --- | --- | --- |
| **Symptom** | **All sites**  **Negative Likelihood Ratio (95% CI)** | **Site 1, 2**  **Negative Likelihood Ratio (95% CI)** | **Site 3**  **Negative Likelihood Ratio (95% CI)** |
| **In 5-18 year olds, impact of the ABSENCE of each symptom on COVID-19 positivity** | | | |
| Fever or chills | 0.91 (0.77-1.07) | 0.95 (0.75-1.20) | 0.85 (0.68-1.07) |
| Sore Throat† | 1.00 (0.83-1.21) | 0.44 (0.13-1.42)* | 1.11 (0.91-1.35) |
| Headache† | 0.74 (0.59-0.93)* | 0.82 (0.43-1.57) | 0.76 (0.60-0.97)* |
| Cough | 0.87 (0.75-1.00) | 0.91 (0.74-1.12) | 0.8 (0.66-0.97)* |
| Nausea, vomiting, or diarrhea | 1.15 (1.05-1.26)* | 1.18 (1.06-1.32)* | 1.17 (1.01-1.36)* |
| Shortness of Breath | 0.99 (0.95-1.03) | 1.01 (0.97-1.05) | 0.99 (0.92-1.06) |
| Nasal Congestion/Rhinorrhea | 0.99 (0.86-1.15) | 1.09 (0.89-1.32) | 0.88 (0.71-1.09) |
| Fatigue§ | 0.43 (0.13-1.38) | 0.43 (0.13-1.38) | # |
| Abdominal Pain† | 1.12 (1.03-1.23)* | 1.05 (0.77-1.44) | 1.15 (1.04-1.27)* |
| Muscle Aches | 0.91 (0.82-0.99)* | 0.98 (0.87-1.09) | 0.86 (0.74-0.99)* |
| Loss of taste or smell | 0.88 (0.82-0.94)* | 0.90 (0.83-0.99)* | 0.87 (0.78-0.96)* |
| Conjunctivitis‡ | 1.00 (0.95-1.05) | 1.00 (0.95-1.05) | # |
| Rash | 1.03 (1.01-1.04)* | 1.03 (1.00-1.05) | 1.02 (0.99-1.05) |
| Self-reported COVID-19 contact exposure | 0.35 (0.28-0.44)* | 0.40 (0.29-0.55)* | 0.30 (0.21-0.42)* |
| **In 0-4 year olds, impact of the ABSENCE of each symptom on COVID-19 positivity** | | | |
| Fever or chills | 0.65 (0.46-0.92)* | 0.81 (0.55-1.19) | 0.60 (0.29-1.20) |
| Cough | 1.15 (0.98-1.35) | 1.39 (1.19-1.63)* | 0.82 (0.60-1.12) |
| Nausea, vomiting, or diarrhea | 1.07 (0.96-1.20) | 1.10 (0.96-1.26) | 1.10 (0.90-1.35) |
| Sore Throat† | 0.94 (0.83-1.08) | 0.93 (0.70-1.23) | 0.96 (0.82-1.12) |
| Headache† | 1.02 (0.96-1.09) | 0.94 (0.78-1.13) | 1.10 (1.01-1.19) |
| Shortness of Breath | 0.98 (0.95-1.02) | 1.01 (0.96-1.03) | 0.96 (0.89-1.05) |
| Nasal Congestion/Rhinorrhea | 0.95 (0.74-1.22) | 1.02 (0.73-1.43) | 0.74 (0.50-1.09) |
| Fatigue§ | 0.91 (0.63-1.31) | 0.91 (0.63-1.31) | # |
| Abdominal Pain† | 1.04 (0.96-1.13) | 1.07 (0.91-1.17) | 1.09 (0.96-1.23) |
| Rash | 1.00 (0.96-1.05) | 1.04 (0.99-1.07) | 0.97 (0.87-1.08) |
| Conjunctivitis‡ | 0.99 (0.94-1.04) | 0.99 (0.94-1.04) | # |
| Muscle Aches | 0.98 (0.93-1.03) | 0.98 (0.91-1.05) | 0.98 (0.90-1.06) |
| Loss of taste or smell | 1.00 (0.98-1.01) | 1.00 (0.96-1.02) | \|\| |
| Self-reported COVID-19 contact exposure | 0.33 (0.23-0.47)* | 0.38 (0.24-0.58)* | 0.28 (0.16-0.50)* |
| *p<0.05  † Includes only children tested at Site 2 and 3; Site 1 symptom screener did not include.  ‡ Includes only children tested at Site 2; Sites 1 and 3 symptom screeners did not include.  § Includes only children tested at Site 1 and 2; Site 3 symptom screener did not include.  \|\| Cannot calculate LR for this group, as all children with this symptom tested COVID-19 negative.  # LR unreportable; symptom not on screener. | | | |
